# Physiological activation of human and mouse bitter taste receptors by bile acids

**DOI:** 10.1038/s42003-023-04971-3

**Published:** 2023-06-07

**Authors:** Florian Ziegler, Alexandra Steuer, Antonella Di Pizio, Maik Behrens

**Affiliations:** Leibniz Institute for Food Systems Biology at the Technical University of Munich, Freising, Germany

**Keywords:** Taste receptors, High-throughput screening

## Abstract

Beside the oral cavity, bitter taste receptors are expressed in several non-gustatory tissues. Whether extra-oral bitter taste receptors function as sensors for endogenous agonists is unknown. To address this question, we devised functional experiments combined with molecular modeling approaches to investigate human and mouse receptors using a variety of bile acids as candidate agonists. We show that five human and six mouse receptors are responsive to an array of bile acids. Moreover, their activation threshold concentrations match published data of bile acid concentrations in human body fluids, suggesting a putative physiological activation of non-gustatory bitter receptors. We conclude that these receptors could serve as sensors for endogenous bile acid levels. These results also indicate that bitter receptor evolution may not be driven solely by foodstuff or xenobiotic stimuli, but also depend on endogenous ligands. The determined bitter receptor activation profiles of bile acids now enable detailed physiological model studies.

## Abstract (web summary)

Using a combined experimental-computational approach, the activation profiles of human and mouse bitter taste receptors, TAS2Rs and Tas2rs, by bile acids is described, suggesting a physiological role of bile acids for taste receptors in non-gustatory tissues.

## Introduction

The mammalian taste system is generally able to distinguish the five basic taste qualities salty, sour, sweet, umami and bitter^1,2^. Among the four different cell types housed in taste buds, the type II cells express the G protein-coupled receptors (GPCRs) mediating sweet, umami and bitter taste^3^. Taste GPCRs are divided into the two subfamilies TAS1Rs and TAS2Rs^4^. The three TAS1R members, TAS1R1, TAS1R2, and TAS1R3, form the heterodimers TAS1R1/TAS1R3 for the functional umami and TAS1R2/TAS1R3 for the functional sweet taste receptor^5–10^. In contrast, the group of TAS2Rs consists of ~25 known functional bitter taste receptors in human, but this number varies considerably among species^11^. Despite the relatively few bitter taste receptors, mammals are able to sense hundreds of bitter tasting compounds^12–14^. This is ensured by the presence of broadly tuned receptors, which can detect high numbers of chemically diverse agonists^12^. In humans, the three broadly tuned receptors TAS2R10^15,16^, TAS2R14^17^ and TAS2R46^14^ recognize together more than half of all tested bitter substances^12^. Narrowly and intermediately tuned bitter taste receptors were shown to have more restricted agonist profiles^12^. A physiological relevance for the observed differences in tuning breadths remains to be determined.

In general, strong bitter taste is perceived as unpleasant and as many noxious substances are known to taste bitter, it was thought to have its main function in warning mammals of the ingestion of toxic substances^18^, although no strict correlation between bitterness and toxicity has been observed^19,20^. Moreover, the expression of bitter taste receptors also in non-gustatory tissues has been confirmed. The detection in tissues like the gastrointestinal tract, the respiratory tract and the heart hints at further biological functions beyond taste^21–23^. Beside the gastrointestinal tract, where the swallowed food compounds may directly activate the expressed bitter taste receptors, research is ongoing to uncover agonists of extra-oral bitter taste receptors. Activation of TAS2Rs in airway epithelial cells for example, was already shown to induce increase in ciliary beat frequency to speed up mucociliary clearance with bacterial quorum-sensing molecules as suggested agonists^22^.

It has long been known, that the body fluid bile containing the endogenously produced compound class of bile acids tastes extremely bitter^24^. In human, bile acids are produced as the primary bile acids cholic acid and chenodeoxycholic acid in the liver from cholesterol as scaffold structure, secreted into the gallbladder after conjugation to taurine or glycine and released into the small intestine in response to food intake as they primarily fulfill nutritional functions, like solubilization of lipophilic food compounds^25–27^. In the intestinal lumen, they are exposed to the gut microbiota, which further modify them to secondary bile acids^28,29^. By reabsorption, bile acids are released into the portal venous blood and transported back to the liver to start the circulation again^30,31^. Only a small proportion of bile acids is excreted by the feces or enters the systemic circulation^32^.

Besides their role in digestion, bile acids are already known to fulfill further physiological functions by activation of receptors like the GPCR TGR5^33^. The stimulation of this receptor by taurolithocholic acid in human macrophages induces the expression of anti-inflammatory cytokine IL-10 and reduces the expression of proinflammatory cytokines, indicating the importance of bile acids in immune responses^34^. Therefore, the question arises if also extra-oral bitter taste receptors can mediate biological functions by stimulation with bile acids.

In fact, recent studies demonstrated the bile acid taurocholic acid representing an agonist for bitter taste receptors of mouse, human and bony fish^11,13^. For bony fish furthermore, chenodeoxycholic acid, deoxycholic acid, glycocholic acid and taurolithocholic acid were shown to activate the T2R02 of *Latimeria chalumnae* with activation threshold concentration for taurolithocholic acid between 0.3 and 1 µM^11^. Up to now, there are no data about the activation thresholds for human receptors when stimulated with bile acids, however, to conclude a physiological function for endogenous bile acids activating non-gustatory bitter taste receptors (periodic) supra-threshold concentrations are essential.

The human metabolome database (HMDB) revealed the detection of several endogenously occurring bile acids and provides quantitative data for almost 30 of them in various body fluids^35^. To determine if they might be relevant for the activation of non-gustatory TAS2Rs, their bitter taste receptor activation profile has to be investigated in more detail. Therefore, we employed a set of eight bile acids that were detected and quantified in the human body^35^ to perform a complete functional characterization of the activation of human and mouse bitter taste receptors. The chosen bile acids covered a broad range, including primary, secondary and tertiary bile acids.

## Results

### Screening of human and mouse bitter taste receptors for their activation by bile acids

**Fig. 1:**
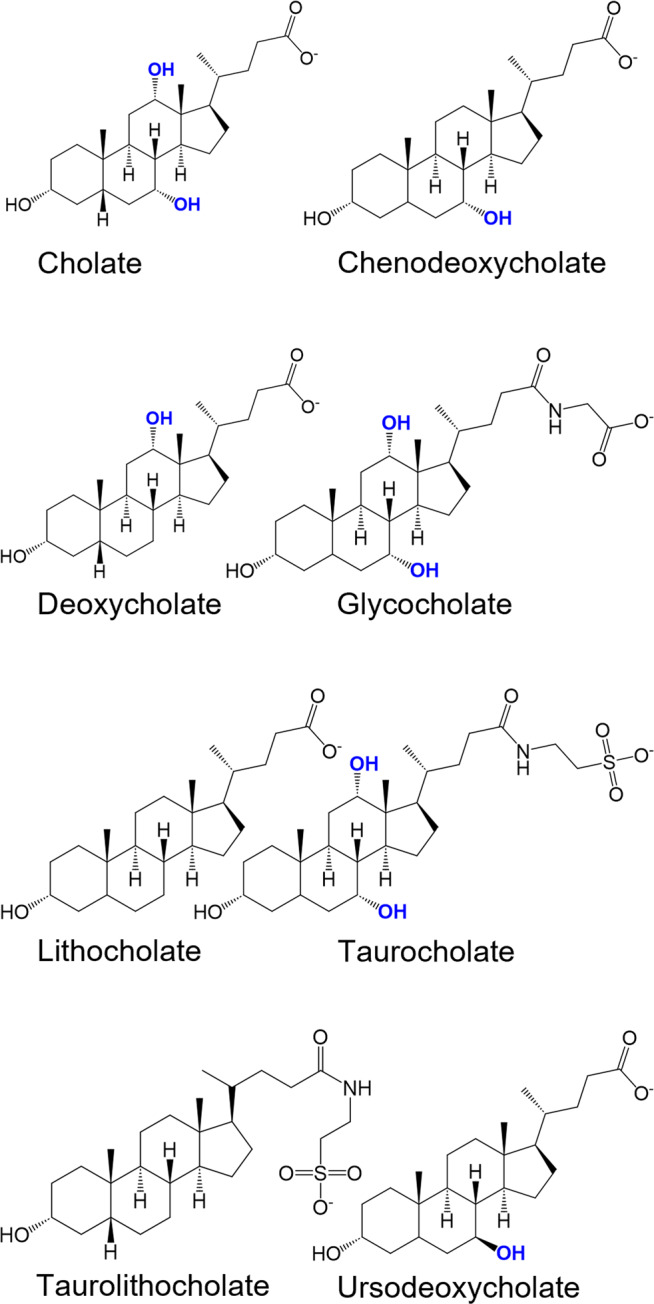
Chemical structures of the investigated bile acids. The structural formulas of the eight bile acids cholic acid, chenodeoxycholic acid, deoxycholic acid, glycocholic acid, lithocholic acid, taurocholic acid, thaurolithocholic acid, and ursodeoxycholic acid in their deprotonated form at physiological conditions (pH ~ 7) are presented. Hydroxyl groups at positions 7 and 12 are highlighted in bold and blue. Structures were generated with ChemDraw software.

To gain a comprehensive insight in the activation profiles of human and mouse bitter taste receptors by bile acids, we tested a set of 8 different bile acids for their activation of 25 human (TAS2R1, −R3, −R4, −R5, −R7, −R8, −R9, −R10, −R13, −R14, −R16, −R19, −R20, −R30, −R31, −R38, −R39, −R40, −R41, −R42, −R43, −R45, −R46, −R50, −R60) and 34 mouse bitter taste receptors (Tas2r102, −r103, −r104, −r105, −r106, −107, −r108, −r109, −r110, −r113, −r114, −r115, −r117, −r118, −r119, −r120, −r121, −r122, −r123, −r124, −r125, −r126, −r129, −r130, −r131, −r134, −r135, −r136, −r137, −r138, −r139, −r140, −r143, −r144). The set of bile acids included the primary bile acids cholic and chenodeoxycholic acid, the secondary bile acids lithocholic and deoxycholic acid, the tertiary bile acid ursodeoxycholic acid, as well as the conjugated bile acids taurolithocholic, glycocholic and taurocholic acid (Fig. 1).

**Fig. 2:**
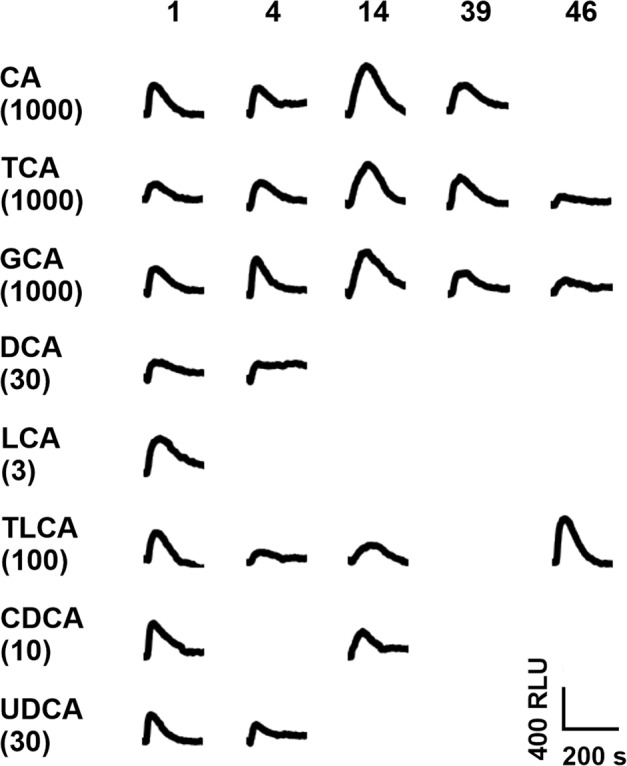
Human bitter taste receptor responses to bile acids. Fluorescence traces of HEK293T-Gα16gust44 cells transiently transfected with expression constructs of the human bitter taste receptors TAS2R1 (1), TAS2R4 (4), TAS2R14 (14), TAS2R39 (39), and TAS2R46 (46). Cells were exposed to cholic acid (CA), taurocholic acid (TCA), glycocholic acid (GCA), deoxycholic acid (DCA), lithocholic acid (LCA), taurolithocholic acid (TLCA), chenodeoxycholic acid (CDCA) and ursodeoxycholic acid (UDCA). Fluorescence changes were measured with an automated fluorometric imaging plate reader (FLIPR^TETRA^). Fluorescence traces are negative control corrected. Applied bile acid concentrations are given in µM in brackets. Only traces of responsive receptors are shown. A scale bar is provided at the bottom right.

By performing a screening using Ca^2+^-imaging assay, we were able to identify 5 human bitter taste receptors responding to bile acids (Fig. 2).

The human bitter taste receptors TAS2R1, TAS2R4, TAS2R14, TAS2R39 and TAS2R46 responded to at least three of the tested bile acids. Of those, TAS2R1 was the least selective as it was stimulated by all eight bile acids. Additionally, TAS2R4 was activated by 6, TAS2R14 by 5, and both TAS2R39 and TAS2R46 by 3 of the bile acids.

**Fig. 3:**
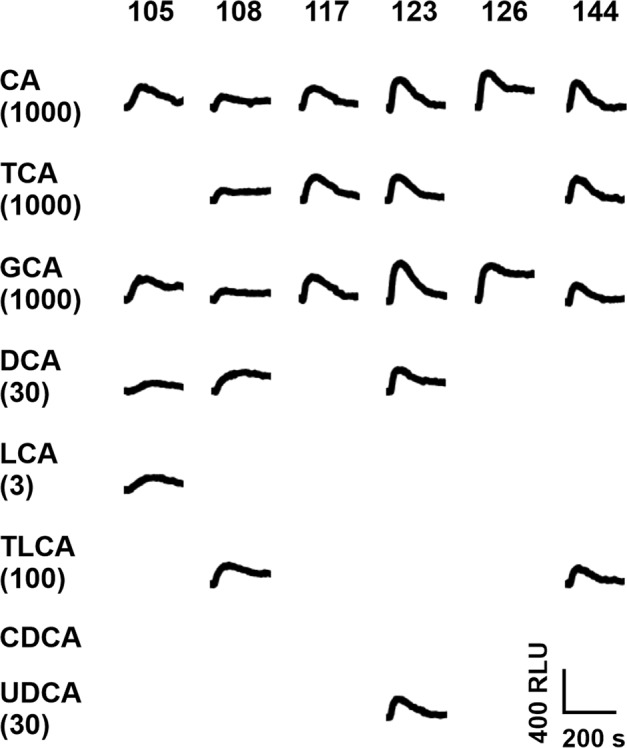
Mouse bitter taste receptor responses to bile acids. Fluorescence traces of HEK293T-Gα16gust44 cells transiently transfected with expression constructs of the mouse bitter taste receptors Tas2r105 (105), Tas2r108 (108), Tas2r117 (117), Tas2r123 (123), Tas2r126 (126), and Tas2r144 (144). Cells were exposed to cholic acid (CA), taurocholic acid (TCA), glycocholic acid (GCA), deoxycholic acid (DCA), lithocholic acid (LCA), taurolithocholic acid (TLCA), chenodeoxycholic acid (CDCA) and ursodeoxycholic acid (UDCA). Fluorescence changes were measured with an automated fluorescence plate reader (FLIPR^TETRA^). Fluorescence traces are negative control corrected. Applied bile acid concentrations are given in µM in brackets. Only traces of responsive receptors are shown. A scale bar is provided at the bottom right.

To assess the potential phylogenetic conservation of the responses observed for the human receptors, we performed the identical screening procedure using mouse Tas2rs (Fig. 3).

For mouse bitter taste receptors Tas2r105, Tas2r108, Tas2r117, Tas2r123, Tas2r126 and Tas2r144 responses to bile acids were observed. Compared to the human receptors, none of them responded to all tested bile acids and CDCA did not activate any of the tested mouse Tas2rs.

### Establishment of dose-response relationships with activated bitter taste receptors

**Fig. 4:**
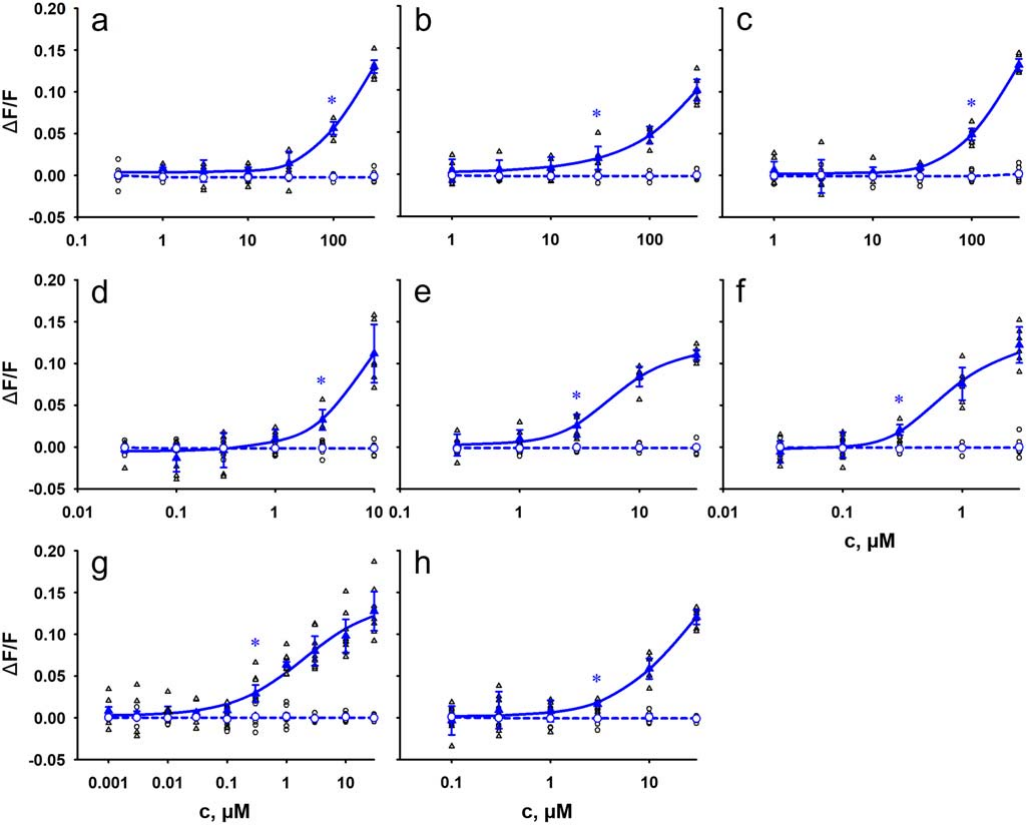
Concentration-response relationships of eight tested bile acids with human TAS2R1. HEK293T-Gα16gust44 cells were transiently transfected with human TAS2R1 (triangle, blue) and an empty vector control (circle, blue). Individual data points are depicted accordingly by black symbols. Receptor activation was recorded by increasing fluorescence intensities upon Ca^2+^ - release using an automated fluorometric imaging plate reader (FLIPR^TETRA^). For dose-response relationships, increasing concentrations of the bile acids cholic acid **a**), taurocholic acid **b**), glycocholic acid **c**), chenodeoxycholic acid **d**), deoxycholic acid **e**), lithocholic acid **f**), taurolithocholic acid **g**) and ursodeoxycholic acid **h**) were applied. The relative fluorescence intensities were mock subtracted and plotted against the bile acid concentration in µM (*n* = 3 biologically independent experiments). Data are presented as the mean ± standard deviation (STD). Beginning statistical significance (*p* < 0.01) is indicated by (*).

To elucidate the potency of bile acids to activate human bitter taste receptors and thus get a first hint if endogenous bile acid concentrations could suffice to activate bitter taste receptors, dose-response relationships were established (Fig. 4, Supplementary Figures 1–4). For this purpose, different concentrations of the bile acids were applied to the cells expressing the receptors. The lowest concentration eliciting a significantly increased fluorescence response (*p* < 0.01) compared to the empty vector control was defined as activation threshold concentration (Table 1). In case receptor saturation was reached with the highest applied bile acid concentration, we further calculated the EC_50_-value (Supplementary Table 1).

**Table 1:** Activation threshold concentrations of bile acids with human bitter taste receptors.

	C_blood_	Max c	TAS2R1	TAS2R4	TAS2R14	TAS2R39	TAS2R46
**Cholic Acid**	0.1−1.7^61, 62^	1000	100	30	300	300	
**Chenodeoxycholic Acid**	0.2−1.8^61, 62^	30	3		30		
**Lithocholic Acid**	0.08−0.33^55, 62^	3	0.3				
**Deoxycholic Acid**	0.33−0.57^55, 63^	30	3	3			
**Taurocholic Acid**	0.1−0.38^61, 63^	1000	100	100	300	300	1000
**Glycocholic Acid**	0.6−0.88^61, 64^	1000	100	30	100	300	1000
**Taurolithocholic Acid**	0.61−1.81^65^	100	0.3	1	0.3		0.3
**Ursodeoxycholic Acid**	0.16^55^	30	3	3			

Presentation of TAS2Rs that were activated by bile acids. Determined threshold concentrations (p < 0.01) for receptor activation and maximum applied bile acid concentrations (Max c) are given in µM. Published bile acid concentration ranges in human blood (c_blood_;in µM) as summarized in the human metabolome database are listed.

In doing so, the secondary bile acid lithocholic acid and its taurine-conjugated form taurolithocholic acid were identified as the most potent bile acids. For lithocholic acid, the determined threshold concentration for the activation of the TAS2R1 was 0.3 µM. Same threshold values were detected for the activation of TAS2R1, TAS2R14, and TAS2R46 by taurolithocholic acid. For TAS2R1, also the EC_50_-values of these two bile acids were in the high nanomolar and low micromolar range, respectively (Supplementary Table 1). In total, the activation threshold concentrations of all tested bile acids varied considerably and ranged between high nanomolar values for the mentioned bile acids and low millimolar values for the activation of TAS2R46 by taurocholic acid and glycocholic acid (Table 1).

**Fig. 5:**
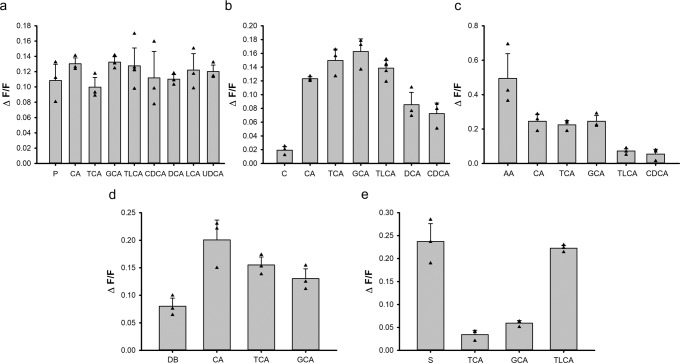
Comparison of the efficacies of bile acids with prototypical TAS2R agonists. Human bitter taste receptors TAS2R1 **a**), TAS2R4 **b**), TAS2R14 **c**), TAS2R39 **d**), and TAS2R46 **e**) activated by highest applied bile acid concentrations (cholic acid (CA), taurocholic acid (TCA), glycocholic acid (GCA), taurolithocholic acid (TLCA), chenodeoxycholic acid (CDCA), deoxycholic acid (DCA), lithocholic acid (LCA), ursodeoxycholic acid (UDCA)) are presented (*n* = 3). For comparison, maximal signal amplitudes (ΔF/F) obtained with control stimuli of the corresponding TAS2Rs were added. The control stimuli were: 1 mM picrotoxinin (P) for TAS2R1, 3 mM colchicine (C) for TAS2R4, 10 µM aristolochic acid (AA) for TAS2R14, 3 mM denatonium benzoate (DB) for TAS2R39 and 10 µM strychnine (S) for TAS2R46. Data are presented as the mean ± standard deviation (STD). Individual data points are depicted by black triangles.

To evaluate the agonistic efficacy of bile acids for human TAS2Rs, we compared the signal amplitudes of well-known TAS2R agonists with bile acids activating TAS2R1, TAS2R4, TAS2R14, TAS2R39 and TAS2R46 (Fig. 5).

In case of TAS2R1, measured efficacies of all 8 bile acids were comparable with the control stimulus picrotoxinin. For TAS2R14, the agonist aristolochic acid elicited responses about twice as high as the most effective bile acids and the response of TAS2R46 to taurolithocholic acid is about equal to that obtained for strychnine. Bile acids responses of TAS2R4 and TAS2R39 were considerably stronger than for the control stimuli colchicine and denatonium benzoate, respectively.

**Fig. 6:**
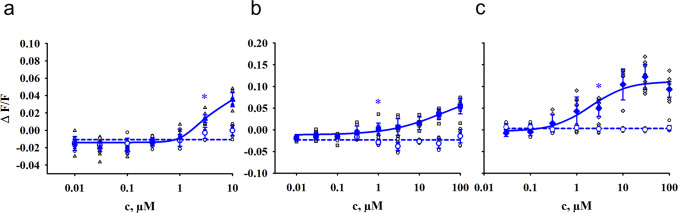
Concentration-response relationships for the activation of mouse Tas2rs. HEK293T-Gα16gust44 cells were transiently transfected with the murine Tas2r105 (triangle, blue), Tas2r108 (square, blue) or Tas2r144 (diamond, blue) and an empty vector control (circle, blue). Individual data points are depicted accordingly by black symbols. Receptor activation was recorded by increasing fluorescence intensities upon Ca^2+^ - release using an automated fluorometric imaging plate reader (FLIPR^TETRA^). For dose-response relationships, increasing concentrations of the bile acids lithocholic acid **a**) and taurolithocholic acid **b**) and **c**) were applied. The relative fluorescence intensities were mock subtracted and plotted against the bile acid concentration in µM (*n* = 3 biologically independent experiments). Data are presented as the mean ± standard deviation (STD). Beginning statistical significance (*p* < 0.01) is indicated by (*).

The same workflow for the evaluation of potencies was applied for the 34 mouse Tas2rs. Again, we calculated activation threshold concentrations (Table 2) and generate dose-response relationships (Fig. 6, Supplementary Figures 5–10).

**Table 2:** Activation of mouse Tas2rs by bile acids.

	Max c (in µM)	Tas2r105	Tas2r108	Tas2r117	Tas2r123	Tas2r126	Tas2r144
**Cholic Acid**	1000	300	100	10	10	100	100
**Chenodeoxycholic Acid**	30						
**Lithocholic Acid**	3	3					
**Deoxycholic Acid**	30	10	10		10		
**Taurocholic Acid**	1000		100	30	100		300
**Glycocholic Acid**	1000	300	100	3	30	100	100
**Taurolithocholic Acid**	100		1				3
**Ursodeoxycholic Acid**	30				10		

Presentation of the mouse Tas2rs that were activated by bile acids. Determined threshold concentrations (p < 0.01) for receptor activation and maximum applied bile acid concentrations (Max c) are given in µM.

As it was already shown for the human receptors, lithocholic acid and taurolithocholic acid are also the most potent bile acids for mouse Tas2rs (Table 2).

By generating dose-response relationships, the threshold concentration for the activation of the Tas2r108 by taurolithocholic acid was determined as 1 µM. With 3 µM the activation threshold concentration of lithocholic acid for Tas2r105 and taurolithocholic acid for Tas2r144 were in a similar range (Fig. 6). Compared to the mouse bitter taste receptors, the human receptors are more sensitive for these two bile acids.

### Predicted binding modes of bile acids within the TAS2R1 binding site

The 3D structure of TAS2R1 was obtained with homology modeling using the recently solved structure of TAS2R46^36^ as a template (sequence identity = 27%). Interestingly, the first solved structures of TAS2R46 suggest a high flexibility of the EC loops, specifically of the ECL2 domain, which is not resolved in the bound state conformation of the receptor, and for which the folding obtained in the unbound states overlaps with the ligand position. The ECL2 connects transmembrane helices 4 and 5 and is diverse in length and composition in currently solved GPCRs^37^. It was demonstrated that docking performance could be insensitive to or even improved by excluding ECL2 from the calculations^16,38,39^ Therefore, because of the uncertainness of the ECL2 folding, we modelled TAS2R1 without the ECL2.

To predict the binding modes of bile acids within the orthosteric TAS2R1 binding site, we ran molecular docking simulations, and generated thirty different poses for each compound. Among all poses, we selected a consensus binding mode, namely the most frequent pose observed for all ligands that is also the best pose for lithocholic acid. The ligands insert into the orthosteric binding site by anchoring to TM3 and TM5. These poses have docking scores that correlate well with activation thresholds, but to further optimize ligand-receptor interactions, the poses were rescored with MM/GBSA minimization. The resulting binding modes were not affected but the scoring improved even more (Table 3, Supplementary Figures 11 and 12), suggesting that the model can capture key ligand-receptor interactions and supporting the assumption that bile acids bind to the orthosteric binding site.

**Table 3:** Docking and MM/GBSA scores of analyzed bile acids within the TAS2R1 binding site.

	Activation thresholds [µM]	Docking scores [kcal/mol]	MM/GBSA dG Bind [kcal/mol]
**Taurolithocholic Acid**	0.30	−5.61	−86.45
**Lithocholic Acid**	0.30	−5.28	−70.11
**Chenodeoxycholic Acid**	3.00	−5.08	−69.75
**Ursodeoxycholic Acid**	3.00	−5.78	−69.60
**Deoxycholic Acid**	3.00	−5.43	−68.36
**Cholic Acid**	100.00	−4.57	−65.75
**Glycocholic Acid**	100.00	−3.36	−65.27
**Taurocholic Acid**	100.00	−3.33	−48.76

**Fig. 7:**
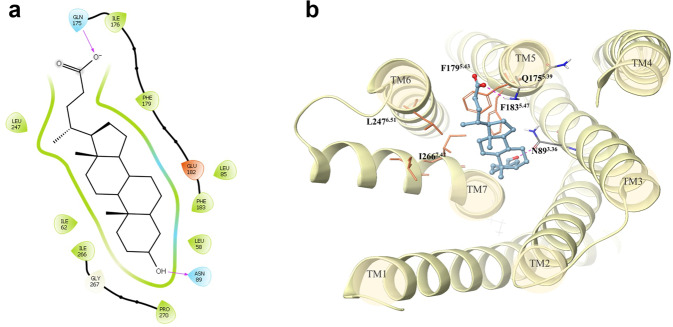
2D and 3D representations of the putative binding mode of lithocholic acid in the TAS2R1 binding site obtained with MM/GBSA refinement. The 2D plot **a**) was generated using the Ligand Interaction Diagram tool available in Maestro (Schrödinger Release 2022-3) showing residues at 4 Å from the ligand. In the 3D representation **b**), the ligand is shown as blue ball&stick, polar residues in CPK-colored sticks and hydrophobic residues as orange sticks. Hydrogen bonds are shown as dashed magenta lines in both representations.

In Fig. 7, we show the 2D and 3D representations of the binding mode of lithocholic acid within TAS2R1. The ligand forms hydrogen bonds with the side chain of N89^3.36^ and the main chain of Q175^5.39^ and it is accommodated in a hydrophobic cavity generated by F179^5.43^, F183^5.47^, L247^6.51^ and I266^7.42^.

The binding modes of all other ligands are reported in Supplementary Figure 11. Lithocholic, chenodeoxycholic, ursodeoxycholic, deoxycholic and cholic acids differ mostly in the presence/absence of hydroxyl groups in position 7 and 12. Interestingly, we found that these hydroxyl groups do not affect the pose of binding but point to F179^5.43^ and F183^5.47^, affecting the hydrophobic interactions observed for lithocholic acid (Fig. 7). Taurolithocholic acid, with a similar activity threshold to lithocholic acid, also misses the hydroxyl groups in positions 7 and 12, suggesting that hydrophobic complementarity is important for receptor binding and activation. Glycocholic and taurocholic acids are the ligands that differ most in the binding mode. The methyl groups of glycocholic acid push the ligand higher in the binding site, we lose the interaction with N89^3.36^, and the ligand anchors instead to Y9 in TM1.

## Discussion

The detection of bitter taste receptors in tissues beside the gustatory system has resulted in an increased interest in the biological role(s) of these receptors in non-gustatory tissues and in the nature and putative origins of the bitter substances that activate the receptors outside the oral cavity. Therefore, one hypothesis is the existence of endogenous agonists^40^ and previous studies already confirmed the activation of human, mouse and bony fish bitter taste receptors by bile acids^11,13^. In general, the group of bile acids is a very complex compound class. As they are released into the small intestine upon food uptake, they are exposed to the gut microbiota. Thereby bile acids are modified and finally a mixture of hundreds of different bile acids is present^28,29^. To get a deeper look into the activation of bitter taste receptors by bile acids, we investigated a diverse group of bile acids, including primary, secondary, and tertiary bile acids. For human and mouse receptors activated by taurocholic acid, we could confirm previously published data according to activation threshold concentrations and EC_50_-values in this study^13^. Besides, we further identified the human TAS2R1, TAS2R14, TAS2R39 and TAS2R46, as well as mouse Tas2r108 as receptors for taurocholic acid. The different outcomes of both studies may occur due to the rather high activation threshold concentrations of this bile acid (30–1000 µM), which is near the highest applied concentration (1000 µM), whereby a weak activation could have been missed in the previous study in which the concentration used for the screening was limited to 300 µM. Moreover, all newly identified taurocholic acid responsive receptors (mouse Tas2r108, human TAS2R1, −R14, −R39, and –R46) exhibited quite high threshold concentrations of 100–1000 µM and, in case of Tas2r108 and TAS2R1, also low signal amplitudes (cf. Figures 2 and 3). For mouse Tas2r105, the lack of taurocholic acid responsiveness reported by Lossow et al.^13^. has been confirmed, although other, previously not tested bile acids, were able to elicit responses of this receptor.

We further compared the efficacies of bile acids with cognate agonists of the identified human TAS2Rs to evaluate the relative strength of TAS2R activation by bile acids. The results demonstrated that for TAS2R1, bile acid responses are on a similar level with the agonist picrotoxinin indicating that bile acids represent full agonists of this receptor. In contrast, the bile acid agonists of TAS2R14 were not able to trigger a response similar to aristolochic acid, which is one of the most efficient agonists for this receptor. Therefore, the tested bile acids represent only partial agonists of TAS2R14. Responses to control stimuli of TAS2R4 and TAS2R39 were found to be less effective than the activating bile acid agonists, indicating that those may represent full agonists for both receptors. As indicated already by the determined dose-response relationships, the TAS2R46 seems to be a receptor specialized to detect distinct bile acids. Beside the differences in activation threshold concentrations, also the efficacy of TLCA is significantly higher than that of TCA and GCA and comparable to the control stimulus strychnine. Hence, TCLA can be judged as another full agonist of this receptor.

As there are several evidences for the functional conservation of bitter taste receptors between species like the metal ion response of human and vampire bats or the overlapping agonist profiles of coelacanth and zebrafish T2R1, we further investigated mouse bitter taste receptors for their bile acid response^11,41,42^. To compare responses of one-to-one orthologous receptors of both species, we consulted the phylogenetic tree that was generated in a former study^13^. Here, we identified the two receptors TAS2R1 and TAS2R39 responding to bile acids, but their corresponding orthologues Tas2r119 and Tas2r139 do not. In contrast, the orthologue of TAS2R4, called Tas2r108 is activated by bile acids. They share common agonists among the tested bile acids, but there are also significant differences. The human receptor is very sensitive to ursodeoxycholic acid with an activation threshold concentration of 3 µM, whereas the mouse receptor does not respond (Table 1, Table 2). We have analyzed the sequences of the orthologous receptors TAS2R4 and Tas2r108. Among the considerable number of differences between the two receptors (98 positions (~33%) are not identical, see Supplementary Figures 13 – 15), only few minor differences occur in positions which have been demonstrated previously to be important for agonist binding (Supplementary Figures 14 and 15). As ursodeoxycholic acid is the only compound in which the C7 hydroxylgroup is positioned above the plane of ring B, we speculate that sterical hindrance between Tas2r108 residues and the C7 hydroxylgroup could be responsible for the lack of activation.

For the remaining responding receptors, no one-to-one orthologues were identified as they are organized in species-specific gene expansion groups. Therefore, we can confirm that a general conservation of the functionality of orthologous receptors between mice and human does not exist, as it was already proposed elsewhere^13^. We did not obtain any response of mouse receptors to CDCA, whereas human TAS2R1 and TAS2R14 were activated by this bile acid. It is known that mice possess an enzyme called Cyp2c70 in the liver, which is converting CDCA into muricholic acid (MCA)^43^. This keeps CDCA levels low, and hence might be the reason why mouse bitter taste receptors did not exhibit responsiveness to CDCA. Whether mouse Tas2rs instead are more specialized to the mouse-specific MCAs has to be clarified.

Furthermore, we demonstrated that the secondary bile acid lithocholic acid and its taurine conjugated form taurolithocholic acid are the most potent tested bile acids in activating human as well as mouse bitter taste receptors. These bile acids are activating the already known bile acid receptor TGR5 with EC_50_ values of 600 nM for lithocholic and 300 nM for taurolithocholic acid^44^. For the human TAS2R1 we measured EC_50_ values of 900 nM and 1.9 µM, respectively, concluding the TGR5 receptor to be slightly more sensitive to lithocholic acid and taurolithocholic acid. Related to these data, it can be assumed that the activation of bitter taste receptors by bile acids is only of biological relevance in tissues, cells or at subcellular localizations where the TGR5 is not expressed, respectively if bile acid concentration is increasing, the activation of bitter taste receptors might be additive to the TGR5 signal. According to literature, there are some tissues with overlapping TGR5 and TAS2R expression profiles, including the small intestine and the testis^45,46^. In particular in testis, TAS2R1 mRNA was detected in late spermatids at quite high levels, whereas TAS2R4 and TAS2R14 mRNAs were found at lower levels in several cell types of the small (TAS2R4: enteroendocrine cells, Paneth cells, goblet cells, enterocytes; TAS2R14: enterocytes) and large (TAS2R4: undifferentiated cells, enterocytes, goblet cells, enteroendocrine cells; TAS2R14: T-cells, enterocytes, undifferentiated cells, goblet cells, enteroendocrine cells) intestine^47^,(https://www.proteinatlas.org/search/TAS2R). It is a matter of future research to elucidate the function of TAS2Rs in these tissues and conclude the interplay between both receptor types. Furthermore, we only tested a subset of the variety of all bile acids. Therefore, we might have missed the best bile acid agonist for human bitter taste receptors, which is more potent to TAS2Rs than to TGR5. However, there are differences in published EC_50_-values of TGR5 activation by bile acids observed and a recent study reported EC_50_-concentrations of 20 µM for LCA and 2.3 µM for TLCA^48^. These results suggest, that occasionally TAS2Rs can be more sensitive to bile acids and fulfill their function independent from TGR5 or that the additive effect is mediated by TGR5.

To answer the question of potential biological relevance, we compared if the quantified concentrations of bile acids in human body fluids listed in the Human Metabolome Database^35^ match with our measured data. As already expected, concentrations in bile are in the millimolar range, for which reason a biological relevance of bitter taste receptors in tissues like the gallbladder, the liver or the small intestine is questionable as these concentrations would lead to a permanent activation of the receptors. A previous study, which showed the absence of the known bile acid bitter taste receptors Tas2r117, Tas2r123 and Tas2r144 in mouse small intestine therefore concluded that the absence of the bile acid-sensitive Tas2rs is due to the fact that such receptors are useless if physiological bile acid concentrations exceed threshold concentrations at all times and hence would constantly signal or remain constantly desensitized^49^. As already mentioned, we were able to identify the Tas2r105, the Tas2r108 and the Tas2r126 as further bile acid bitter taste receptors. Published expression data reveals high intestinal expression levels of the Tas2r108 and the Tas2r126^49^. For human TAS2R expression in the small intestine, it is reported that TAS2R4, TAS2R14, TAS2R39 and TAS2R46, which are all responding to bile acids, are expressed in jejunal crypts. In this context, some functions of these receptors in the small intestine are suggested. The activation of TAS2R4 by taurocholic acid was reported to increase the release of molecules that have a positive impact on *E. coli* growth^50^. Therefore, the ingestion of food, which results in the release of bile acids into the small intestine may have positive effects on *E. coli* growth and consequently for the process of digestion. Furthermore, activation of TAS2R14 in a human colorectal cancer cell line is supposed to result in increased GDF15 levels, which is involved in several biological functions like anti-inflammatory and apoptotic pathways^50–52^. To clarify, if these receptors play a role in bile acid detection in the small intestine, further research is necessary.

Beside high concentrations in bile, blood serum bile acid levels increase from 0.2 – 0.7 µM to 4 – 5 µM postprandial^53,54^ and for lithocholic acid, which is one of the most potent identified bile acids, a serum concentration of 0.33 µM was measured previously in healthy children subjects^55^. As blood is the main transporting unit in the body, this bile acid will be distributed in concentrations that were shown to be sufficient to activate the human TAS2R1, which is highly expressed in the human brain and testis^46^, in particular in late spermatids ^47^,(https://www.proteinatlas.org/search/TAS2R). It was further shown that the lithocholic acid serum concentration is decreased in children with cystic fibrosis and it is known that men with cystic fibrosis go later through puberty than healthy subjects^55,56^. As brain and testis are important players in puberty a role of bile acids in development from child to adult is conceivable, but further research is required.

Interestingly, the potency of activation of the human TAS2R1 seemed to depend a lot on the presence of hydroxyl groups at positions 7 and 12 of the steroid scaffold structure in our experiments. Docking simulations of bile acids investigated in this paper highlighted the main interactions established with TAS2R1. We found that hydroxyl groups at positions 7 and 12 can affect hydrophobic interactions between the ligands and two hydrophobic patches in the receptor binding site: one made by Leu85^3.32^, Phe183^5.47^, and Phe179^5.43^ and the other one made by Ile266^7.42^ and Leu247^6.51^. We also suggest that the H-bond between the ligands and N89^3.36^ is highly important for the ligand-receptor interaction, and it is supposed to be a key interaction for receptor selectivity. In fact, position 3.36 is highly conserved among the investigated TAS2Rs, but the residue in the close position 3.32 can influence the access to this interaction. In TAS2R1, a leucine occupies this position, but we have bulkier residues (F or W) in TAS2R4, TAS2R14, TAS2R39 and TAS2R46. This difference causes a change of pose in other receptors (we report the predicted binding mode for taurolithocholic acid within the TAS2R46 binding site in Supplementary Figure 16).

In conclusion, we were able to show the activation profile of human and mouse bitter taste receptors by bile acids. We identified five human and six mouse receptors, which are responsive to subsets of tested bile acids. Comparing the determined activation threshold concentrations with physiological bile acid concentrations in the human body, this compound class is very promising as endogenous agonists of bitter taste receptors. The comparative investigation of primary cell lines, intestinal organoids, or mouse models derived from TGR5-knockout^57^ and wildtype mice can provide further insights into the activation mechanism and downstream signaling of bitter taste receptors activated by bile acids. It is a future task to experimentally clarify, the exact biological functions of bitter taste receptor activation by bile acids.

## Methods

### Bile acids

Functional characterization of 25 human^12^ and 34 out of 35 mouse bitter taste receptors^13^ was performed using a set of 8 different bile acids, including the primary bile acids cholic (Calbiochem, San Diego, United States) and chenodeoxycholic acid (Sigma-Aldrich, Steinheim, Germany), the secondary bile acids lithocholic (AcrosOrganics, Geel, Belgium) and deoxycholic acid (Sigma-Aldrich, Steinheim, Germany), the conjugated bile acids taurocholic (Biochemika), taurolithocholic (Sigma-Aldrich, Steinheim, Germany), glycocholic acid (Sigma-Aldrich, Steinheim, Germany), as well as the tertiary bile acid ursodeoxycholic acid (Alfa Aesar, Kandel, Germany). This set of bile acids was chosen because of their commercial availability in high purities, their diversity within the class of bile acids and their previous detection and quantitation in human blood. Stock solutions were prepared in DMSO. For the prevention of unspecific cellular responses, the stock solutions are diluted in the assay buffer C1 (130 mM NaCl, 10 mM HEPES pH 7.4, 5 mM KCl, 2 mM CaCl_2_, 0.18 % glucose) to reduce the DMSO concentration to a maximum of 0.5 % in the final experiments. Maximal applied concentrations are due to solubility problems or receptor-independent artefacts during measurement at high bile acid concentrations (Table 1, Table 2)^12,58^.

### Cell lines

As basal growth medium for the HEK293T-Gα16gust44 cell line^17,59^ served Dulbecco’s modified eagle medium (DMEM) (Thermo Fisher Scientific, Darmstadt, Germany) supplemented with 10 % fetal bovine serum (Sigma-Aldrich, Steinheim, Germany), 2 mM L-glutamine (Sigma-Aldrich, Steinheim, Germany), 100 units/ml penicillin (Sigma Aldrich, Steinheim, Germany) and 100 µg/ml streptomycin (Sigma Aldrich, Steinheim, Germany). Growth conditions were 37 °C, 5 % CO_2_ and saturated air-humidity^60^.

### Transient transfection

HEK293T-Gα16gust44 cells were seeded on poly-D-lysine (10 µg/ml) coated 96-well plates to reach a confluence of 40-60 % the next day. For transient transfection 150 ng of plasmid DNA containing the receptor of interest and 0.3 µl lipofectamine 2000 (Thermo Fisher Scientific, Darmstadt, Germany) per well were used and transfection took place according to the manual of lipofectamine 2000. Mouse Tas2r116 was excluded because successful cloning was not possible in a previous work^13^. The empty vector DNA (mock) was transfected as negative control^14,41^.

### Calcium imaging assay

Cells were loaded using the calcium-sensitive fluorescent dye Fluo-4-AM (Abcam, Cambridge, Great Britain) in the presence of 2.5 mM probenecid (Sigma-Aldrich, Steinheim, Germany) the day after transfection^13,41^. 1 h after loading, cells were washed with C1 buffer using a BioTek Cell Washer, incubated in the dark for half an hour and washed again. For automated agonist application and measurement of fluorescence changes, a FLIPR^TETRA^ device (Molecular Devices, San José, United States) was used. Viability of cells was tested by application of 100 nM Somatostatin 14 (Bachem, Bubendorf, Switzerland)^42^.

### Data analysis

Measured data were negative control corrected by subtracting the signal of the mock-transfected cells and exported to Microsoft Excel using the FLIPR software ScreenWorks 4.2. In Microsoft Excel software, standardization of maximum fluorescence intensities to the basal fluorescence and normalization to the buffer-only control was done to calculate the relative fluorescence changes (ΔF/F).

### Statistics and reproducibility

Initial screening experiments performed in duplicate wells were confirmed by at least one replication and representative traces were selected for display. All dose-response relationships were determined by three independent experiments (biological replicates) performed in duplicates (technical replicates). Threshold concentrations, defined as lowest substance concentrations leading to statistically significant elevated fluorescence changes in receptor-transfected cells compared with empty vector-transfected cells, were determined using SigmaPlot with Student’s t-test to evaluate statistical significance (*p* < 0.01).

### Molecular modeling

2D structures of bile acids investigated in this work were downloaded from PubChem. Ligprep (Schrödinger Release 2022-3: LigPrep, Schrödinger, LLC, New York, NY, 2022) was used to generate 3D structures and protonation states of all ligands at pH 7 ± 1.

The currently released receptor structure of TAS2R46 (PDB ID: 7XP6) was used as a template for modeling the structures of TAS2R1, -R4, -R14, and -R39 using Prime (Schrödinger Release 2022-3). The sequence identities between TAS2R1, -R4, -R14, and –R39 and the template are 27%, 24%, 43%, and 25%, respectively. All models are available at https://github.com/dipizio/TAS2R-models.

Glide Standard Precision (Schrödinger Release 2022-3) was used for docking studies on TAS2R1. The receptor binding site was prepared using the “Receptor Grid Generation” tool, the grid box was the centroid of the ligand in the experimental structure of TAS2R46. We saved 30 poses per ligand. The docking pose of lithocholic acid with the lowest Glide score was used as a selection filter for the docking poses of all bile acids. MM/GBSA minimization (Prime, Schrödinger, LLC, New York, NY, USA, 2022) was used to rescore the poses. The same procedure was applied to predict the binding mode of taurolithocholic acid within the TAS2R46 binding site. The 2D and 3D representations of lithocholic acid/TAS2R1 binding mode were generated with Maestro 13.2 (Schrödinger Release 2022-3).

### Reporting summary

Further information on research design is available in the Nature Portfolio Reporting Summary linked to this article.

## Supplementary information

Supplementary Information

Description of Additional Supplementary Files

Supplementary Data 1

Supplementary Data 2

Supplementary Data 3

Reporting Summary

## Data Availability

All data of this study are provided in the main text and supplementary information. Source data for graphs shown in Figs. 2 to 6 are provided as Supplementary data files (Supplementary data 1 for Figs. 2 and 3; Supplementary data 2 for Figs. 4 and 6; Supplementary data 3 for Fig. 5).

## Code availability

Receptor models and docking results are available at https://github.com/dipizio/TAS2R-models.
