# Supplementary material for: Physiological activation of human and mouse bitter taste receptors by bile acids
Source: Commun Biol. 2023 Jun 7;6:612. doi: 10.1038/s42003-023-04971-3 (PMC10247784; doi:10.1038/s42003-023-04971-3)
Supplement: Supplementary file 1 — Supplementary Information [file 42003_2023_4971_MOESM1_ESM.pdf]

Supplementary Information

Physiological activation of human and mouse bitter taste receptors by bile acids

Florian Ziegler, Alexandra Steuer, Antonella Di Pizio and Maik Behrens*

Leibniz Institute for Food Systems Biology at the Technical University of Munich, Freising, Germany.

*Send correspondence to: Maik Behrens, Leibniz Institute for Food Systems Biology at the Technical University of Munich, Lise-Meitner Str. 34, 85354 Freising, Germany. E-mail: m.behrens.leibniz-lsb@tum.de

Supplementary Figures

Supplementary Figure 1: Concentration-response relationships of the activation of the human TAS2R4 by bile acids

Supplementary Figure 2: Concentration-response relationships of the activation of the human TAS2R14 by bile acids

Supplementary Figure 3: Concentration-response relationships of the activation of the human TAS2R39 by bile acids

Supplementary Figure 4: Concentration-response relationships of the activation of the human TAS2R46 by bile acids

Supplementary Figure 5: Concentration-response relationships of the activation of the murine Tas2r105 by bile acids

Supplementary Figure 6: Concentration-response relationships of the activation of the murine Tas2r108 by bile acids

Supplementary Figure 7: Concentration-response relationships of the activation of the murine Tas2r117 by bile acids

Supplementary Figure 8: Concentration-response relationships of the activation of the murine Tas2r123 by bile acids

Supplementary Figure 9: Concentration-response relationships of the activation of the murine Tas2r126 by bile acids

Supplementary Figure 10: Concentration-response relationships of the activation of the murine Tas2r144 by bile acids

Supplementary Figure 11: 3D representations of the putative binding modes of bile acids in the TAS2R1 binding site obtained with MM/GBSA refinement.

Supplementary Figure 12: Correlation plots for all investigated ligands.

Supplementary Figure 13: Amino acid sequence identities of human and mouse bile acid-sensitive receptors.

Supplementary Figure 14: Amino acid sequence alignment of human and mouse bile acid-sensitive receptors.

Supplementary Figure 15: WebLogo depiction of the amino acid sequence alignment of bile acid-sensitive human and mouse bitter taste receptors.

Supplementary Figure 16: 3D representation of the putative taurolithocholic acid binding mode in the TAS2R46 binding site obtained with MM/GBSA refinement.

Supplementary Table

Supplementary Table 1: Calculated EC_{50}-values of human and murine bitter taste receptors activated by bile acids

Supplementary Figures

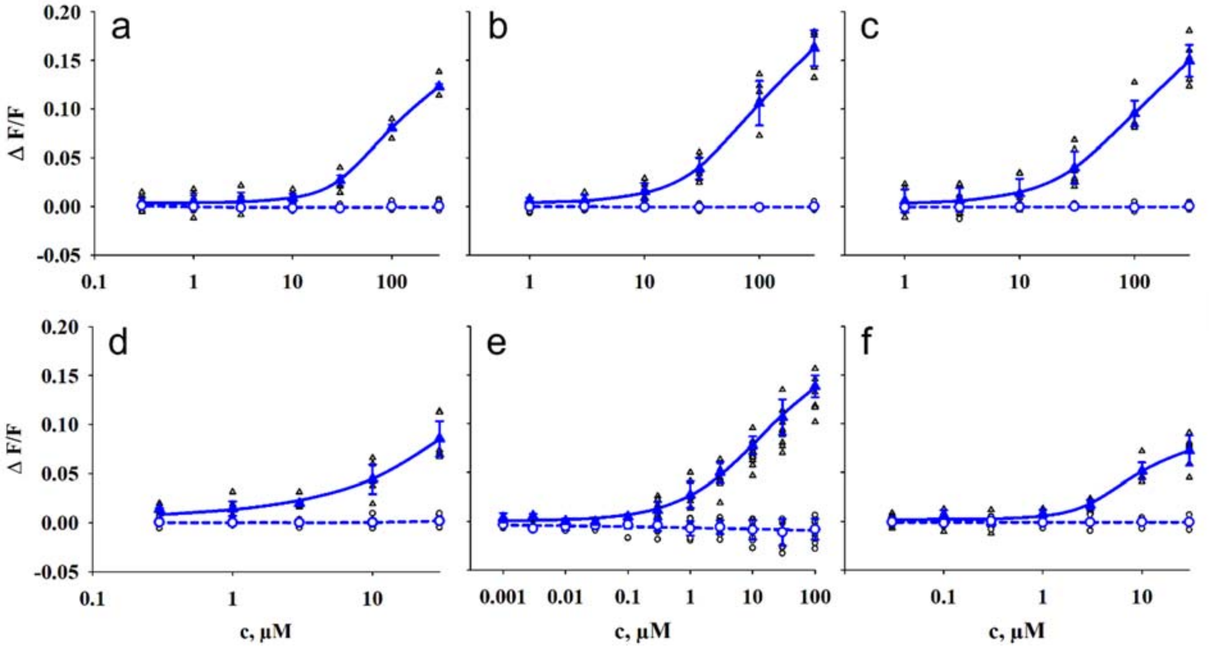

Supplementary Figure 1: Concentration-response relationships of the activation of the human TAS2R4 by bile acids. HEK293T-Gα16gust44 cells were transiently transfected with the human TAS2R14 (triangle, blue) and an empty vector control (circle, blue). Individual data points are depicted accordingly by black symbols. Receptor activation was measured by increasing fluorescence intensities upon Ca^{2+} - release using an automated fluorometric imaging plate reader (FLIPR^{TETRA}). For dose-response relationships, different concentrations of the bile acids cholic acid a), glycocholic acid b), taurocholic acid c), deoxycholic acid d), taurolithocholic acid e) and ursodeoxycholic acid f) were applied. The relative fluorescence intensities were mock subtracted and plotted against the bile acid concentration in μM (n ≥ 3 biologically independent experiments). Data are presented as the mean ± standard deviation (STD).

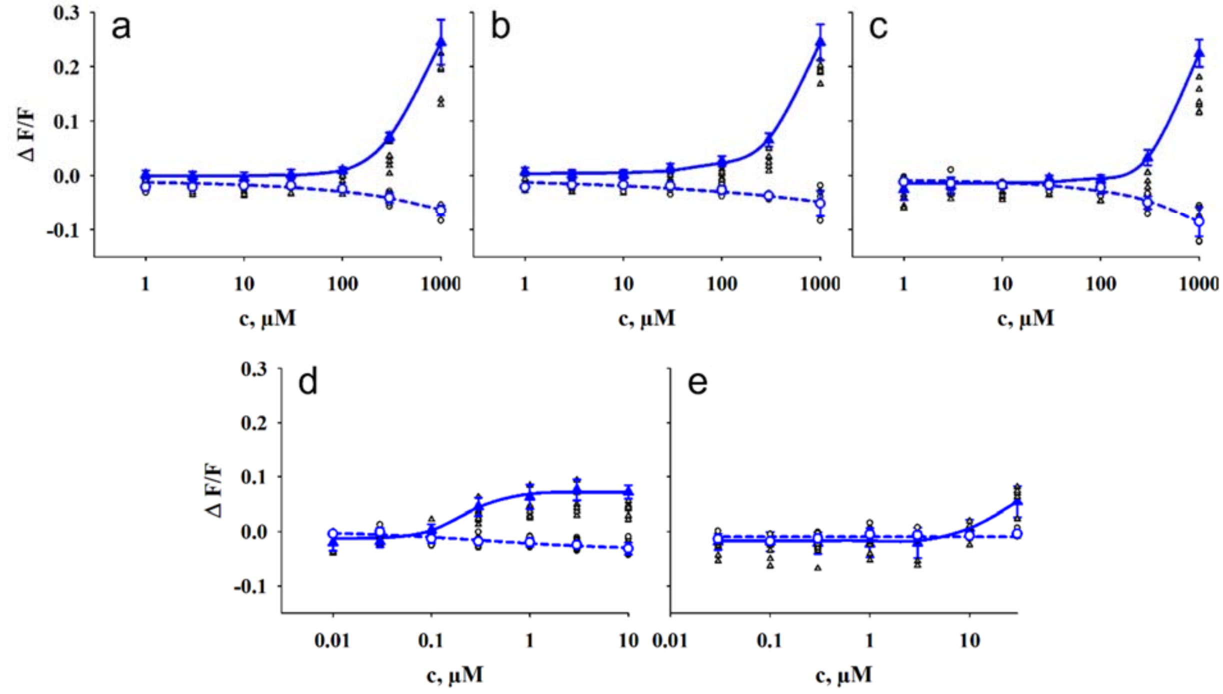

Supplementary Figure 2: Concentration-response relationships of the activation of the human TAS2R14 by bile acids. HEK293T-Gα16gust44 cells were transiently transfected with the human TAS2R14 (triangle, blue) and

an empty vector control (circle, blue). Individual data points are depicted accordingly by black symbols. Receptor activation was measured by increasing fluorescence intensities upon Ca^{2+} - release using an automated fluorometric imaging plate reader (FLIPR^{TETRA}). For dose-response relationships, different concentrations of the bile acids cholic acid a), glycocholic acid b), taurocholic acid c), taurolithocholic acid d) and chenodeoxycholic acid e) were applied. The relative fluorescence intensities were mock subtracted and plotted against the bile acid concentration in µM (n ≥ 3 biologically independent experiments). Data are presented as the mean ± standard deviation (STD).

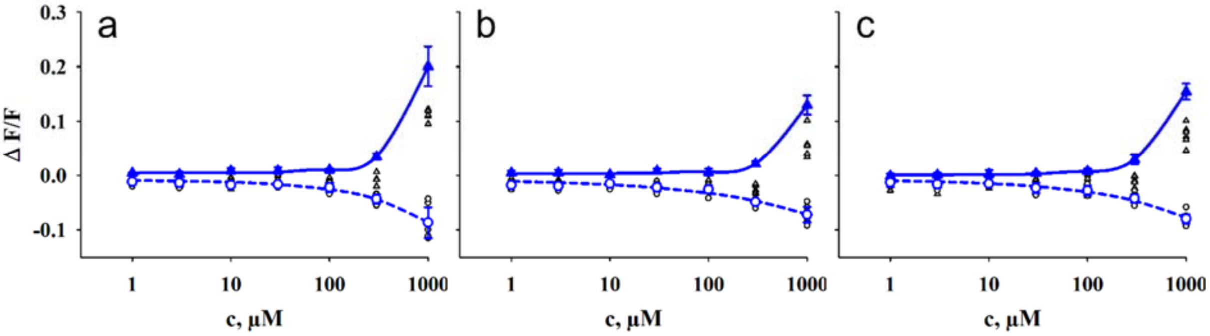

Supplementary Figure 3: Concentration-response relationships of the activation of the human TAS2R39 by bile acids. HEK293T-Gα16gust44 cells were transiently transfected with the human TAS2R39 (triangle, blue) and an empty vector control (circle, blue). Individual data points are depicted accordingly by black symbols. Receptor activation was measured by increasing fluorescence intensities upon Ca^{2+} - release using an automated fluorometric imaging plate reader (FLIPR^{TETRA}). For dose-response relationships, different concentrations of the bile acids cholic acid a), glycocholic acid b) and taurocholic acid c) were applied. The relative fluorescence intensities were mock subtracted and plotted against the bile acid concentration in µM (n ≥ 3 biologically independent experiments). Data are presented as the mean ± standard deviation (STD).

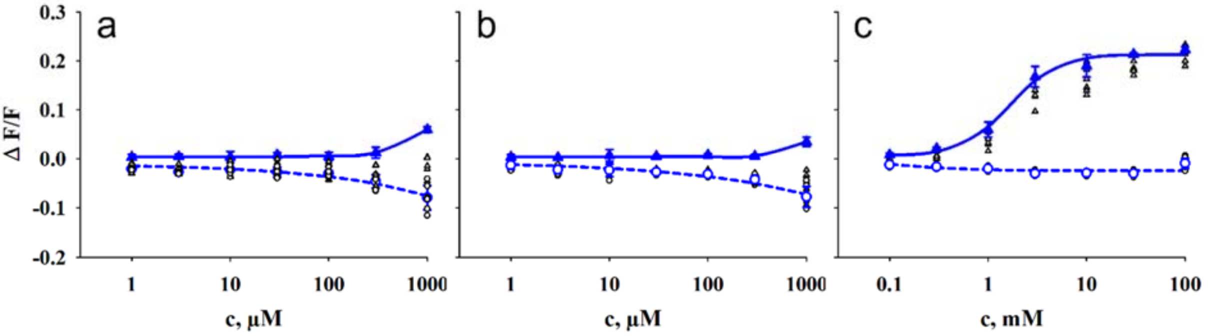

Supplementary Figure 4: Concentration-response relationships of the activation of the human TAS2R46 by bile acids. HEK293T-Gα16gust44 cells were transiently transfected with the human TAS2R46 (triangle, blue) and an empty vector control (circle, blue). Individual data points are depicted accordingly by black symbols. Receptor activation was measured by increasing fluorescence intensities upon Ca^{2+} - release using an automated fluorometric imaging plate reader (FLIPR^{TETRA}). For dose-response relationships, different concentrations of the bile acids glycocholic acid a), taurocholic acid b) and taurolithocholic acid c) were applied. The relative fluorescence intensities were mock subtracted and plotted against the bile acid concentration in µM (n ≥ 3 biologically independent experiments). Data are presented as the mean ± standard deviation (STD).

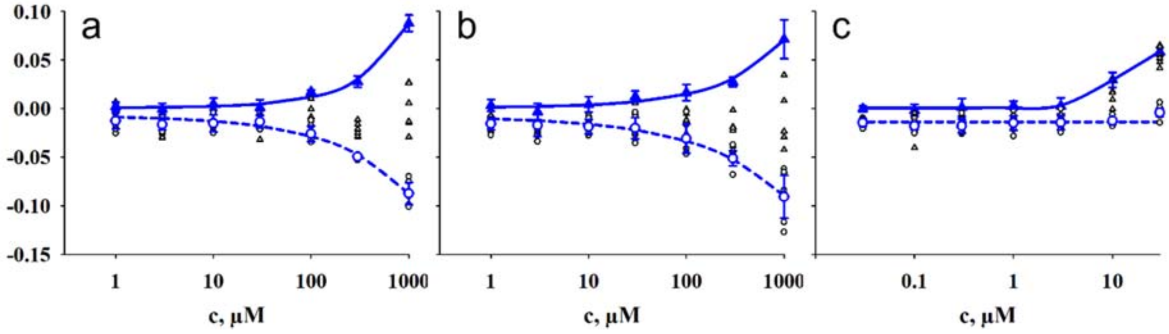

Supplementary Figure 5: Concentration-response relationships of the activation of the murine Tas2r105 by bile acids. HEK293T-Gα16gust44 cells were transiently transfected with the human Tas2r105 (triangle, blue) and an empty vector control (circle, blue). Individual data points are depicted accordingly by black symbols. Receptor activation was measured by increasing fluorescence intensities upon Ca^{2+} - release using an automated fluorometric imaging plate reader ($FLIPR^{TETRA}$). For dose-response relationships, different concentrations of the bile acids cholic acid a), glycocholic acid b) and deoxycholic acid c) were applied. The relative fluorescence intensities were mock subtracted and plotted against the bile acid concentration in μM (n ≥ 3 biologically independent experiments). Data are presented as the mean ± standard deviation (STD).

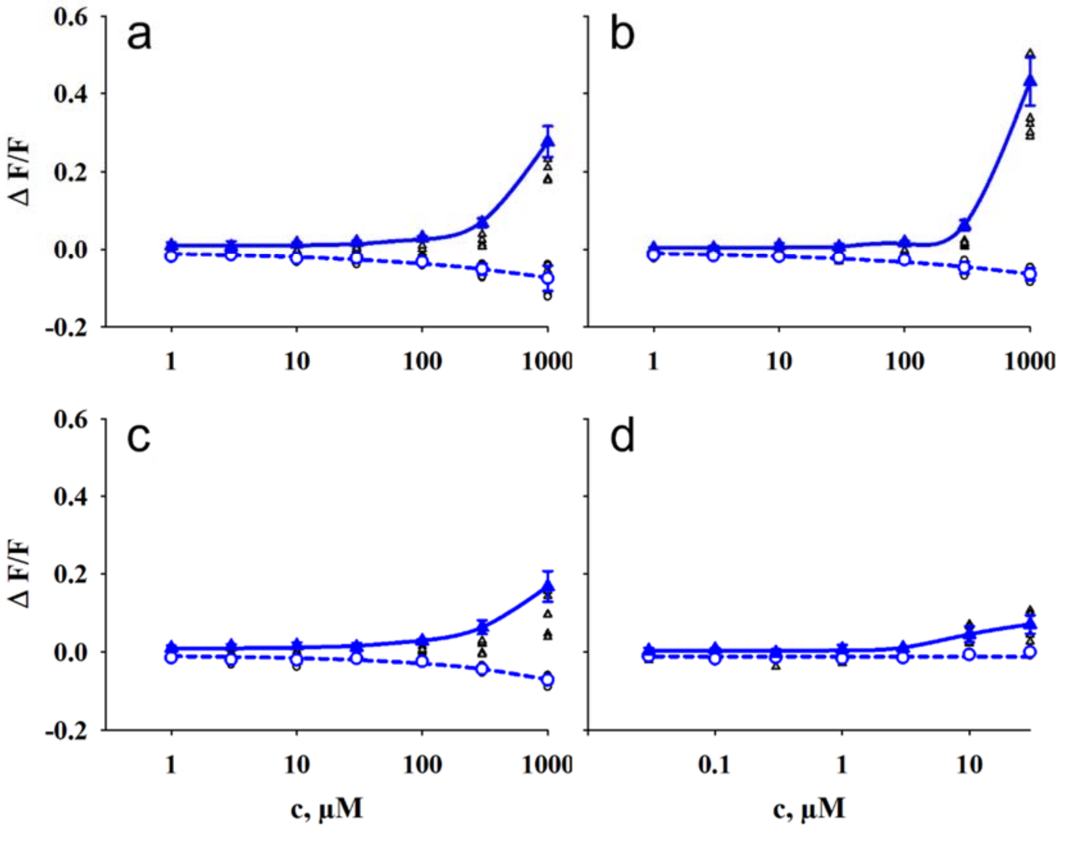

Supplementary Figure 6: Concentration-response relationships of the activation of the murine Tas2r108 by bile acids. HEK293T-Gα16gust44 cells were transiently transfected with the human Tas2r108 (triangle, blue) and an empty vector control (circle, blue). Individual data points are depicted accordingly by black symbols. Receptor activation was measured by increasing fluorescence intensities upon Ca^{2+} - release using an automated fluorometric imaging plate reader ($FLIPR^{TETRA}$). For dose-response relationships, different concentrations of the bile acids cholic acid a), glycocholic acid b), taurocholic acid c) and deoxycholic acid d) were applied. The relative fluorescence intensities were mock subtracted and plotted against the bile acid concentration in μM (n ≥ 3 biologically independent experiments). Data are presented as the mean ± standard deviation (STD).

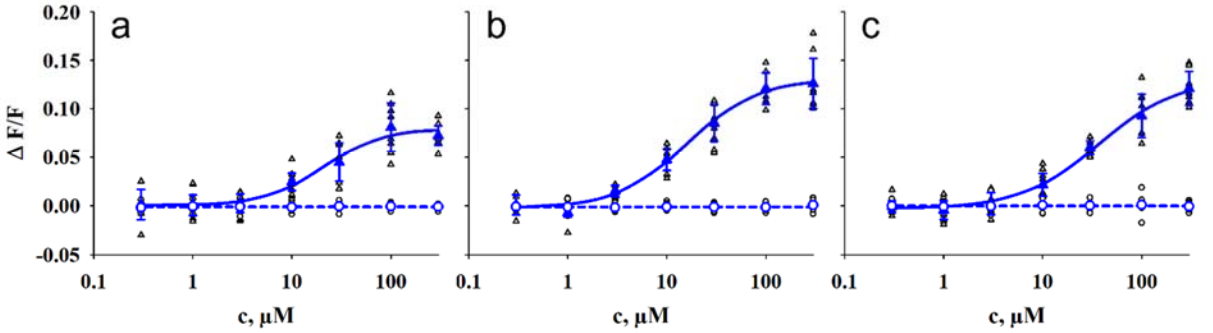

Supplementary Figure 7: Concentration-response relationships of the activation of the murine Tas2r117 by bile acids. HEK293T-Gα16gust44 cells were transiently transfected with the human Tas2r117 (triangle, blue) and an empty vector control (circle, blue). Individual data points are depicted accordingly by black symbols. Receptor activation was measured by increasing fluorescence intensities upon Ca^{2+} - release using an automated fluorometric imaging plate reader (FLIPR^{TETRA}). For dose-response relationships, different concentrations of the bile acids cholic acid a), glycocholic acid b) and taurocholic acid c) were applied. The relative fluorescence intensities were mock subtracted and plotted against the bile acid concentration in µM (n ≥ 3 biologically independent experiments). Data are presented as the mean ± standard deviation (STD).

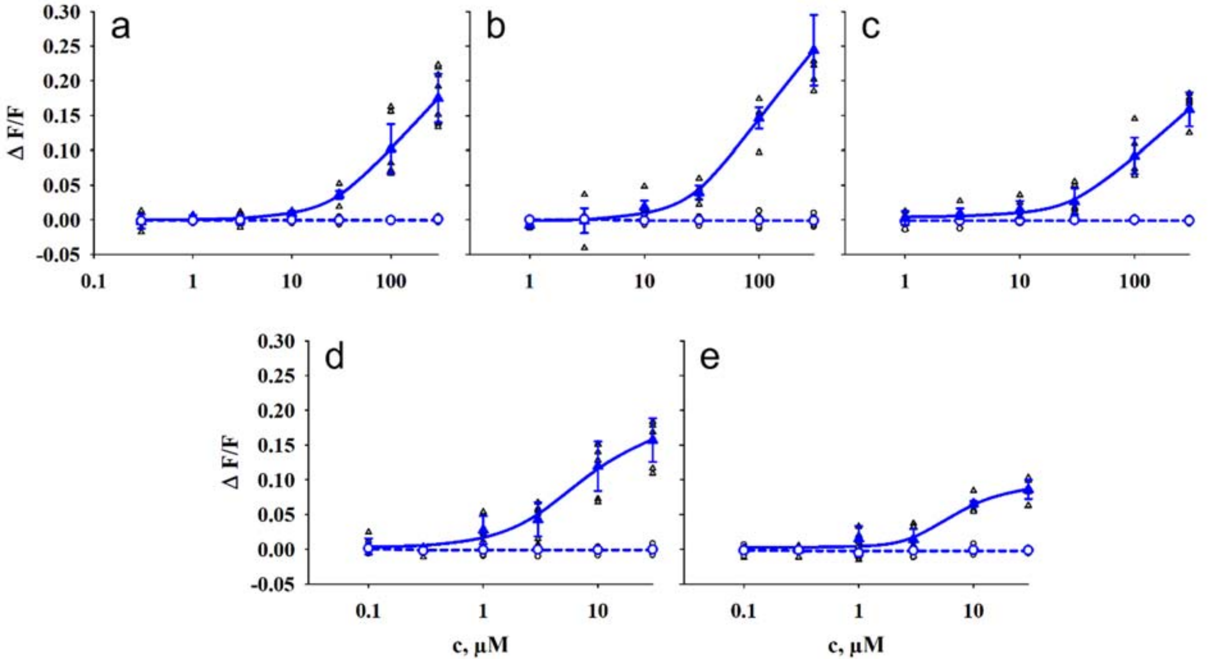

Supplementary Figure 8: Concentration-response relationships of the activation of the murine Tas2r123 by bile acids. HEK293T-Gα16gust44 cells were transiently transfected with the human Tas2r123 (triangle, blue) and an empty vector control (circle, blue). Individual data points are depicted accordingly by black symbols. Receptor activation was measured by increasing fluorescence intensities upon Ca^{2+} - release using an automated fluorometric imaging plate reader (FLIPR^{TETRA}). For dose-response relationships, different concentrations of the bile acids cholic acid a), glycocholic acid b), taurocholic acid c), deoxycholic acid d) and ursodeoxycholic acid e) were applied. The relative fluorescence intensities were mock subtracted and plotted against the bile acid concentration in µM (n ≥ 3 biologically independent experiments). Data are presented as the mean ± standard deviation (STD).

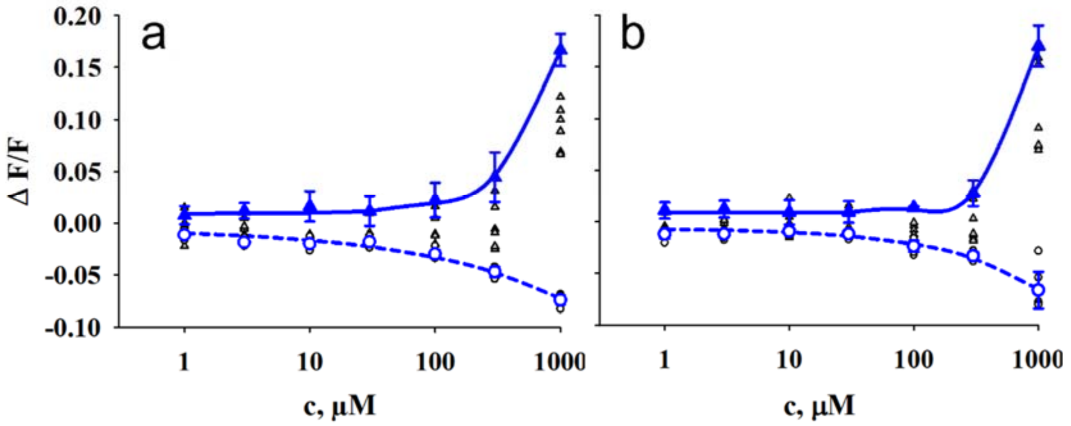

Supplementary Figure 9: Concentration-response relationships of the activation of the murine Tas2r126 by bile acids. HEK293T-Gα16gust44 cells were transiently transfected with the human Tas2r126 (triangle, blue) and an empty vector control (circle, blue). Individual data points are depicted accordingly by black symbols. Receptor activation was measured by increasing fluorescence intensities upon Ca^{2+} - release using an automated fluorometric imaging plate reader (FLIPR^{TETRA}). For dose-response relationships, different concentrations of the bile acids cholic acid a) and glycocholic acid b) were applied. The relative fluorescence intensities were mock subtracted and plotted against the bile acid concentration in µM (n ≥ 3 biologically independent experiments). Data are presented as the mean ± standard deviation (STD).

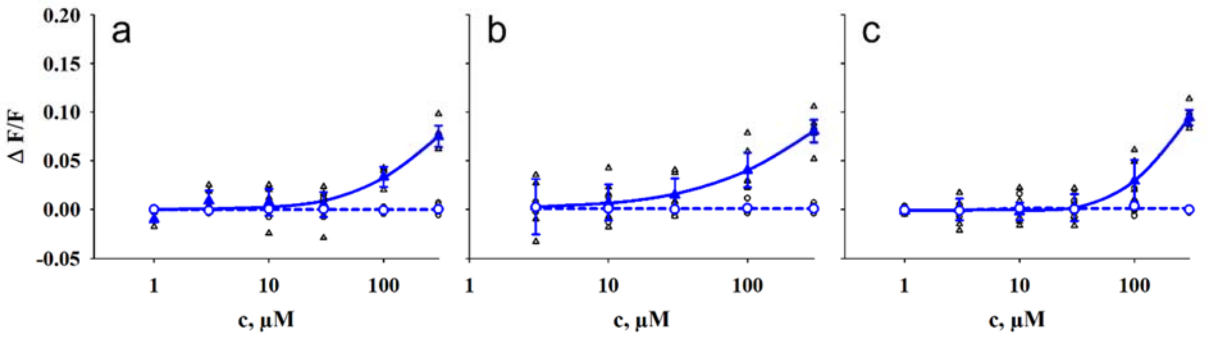

Supplementary Figure 10: Concentration-response relationships of the activation of the murine Tas2r144 by bile acids. HEK293T-Gα16gust44 cells were transiently transfected with the human Tas2r144 (triangle, blue) and an empty vector control (circle, blue). Individual data points are depicted accordingly by black symbols. Receptor activation was measured by increasing fluorescence intensities upon Ca^{2+} - release using an automated fluorometric imaging plate reader (FLIPR^{TETRA}). For dose-response relationships, different concentrations of the bile acids cholic acid (a), glycocholic acid (b) and taurocholic acid (c) were applied. The relative fluorescence intensities were mock subtracted and plotted against the bile acid concentration in µM (n ≥ 3 biologically independent experiments). Data are presented as the mean ± standard deviation (STD).

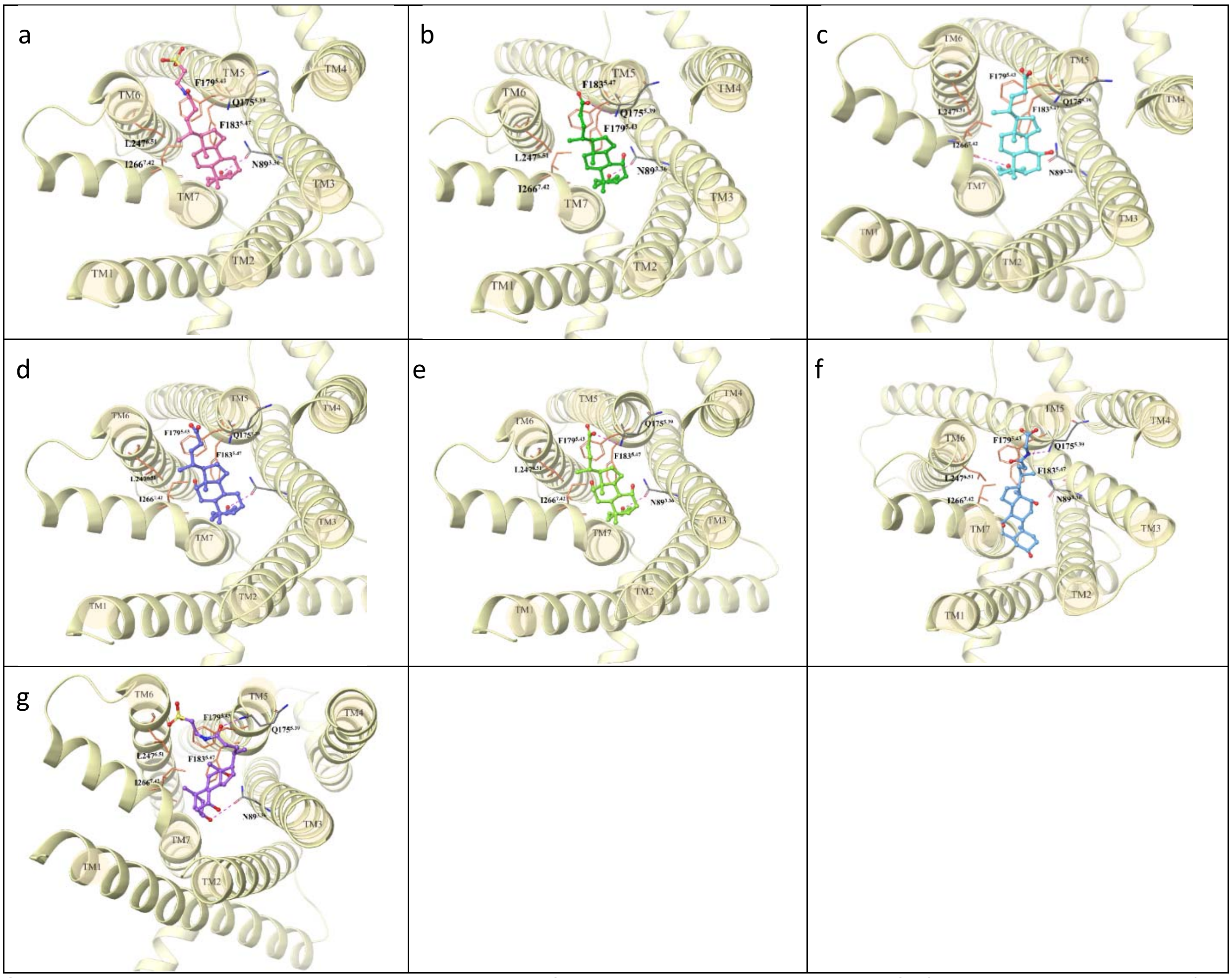

Supplementary Figure 11: 3D representations of the putative binding modes of a) taurolithocholic acid, b) chenoxydecholic acid, c) ursodeoxycholic acid, d) deoxycholic acid, e) cholic acid, f) glycocholic acid, g) taurocholic acid in the TAS2R1 binding site obtained with MM/GBSA refinement.

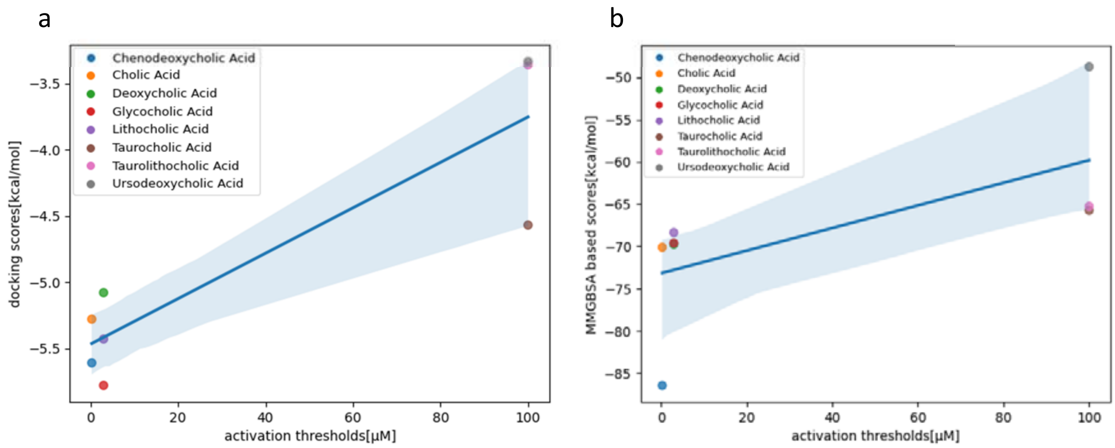

Supplementary Figure 12: Correlation plots of a) docking scores vs. activation thresholds and b) MM/GBSA scores vs. activation thresholds for all investigated ligands.

	TAS2R14	Tas2r123	TAS2R46	Tas2r117	Tas2r105	Tas2r126	TAS2R1	TAS2R39	Tas2r144	TAS2R4	Tas2r108
TAS2R14		44,15	42,38	41,12	30,82	22,64	24,93	23,33	20,52	20,54	21,43
Tas2r123	44,15		38,46	37,54	28,49	24,72	23,21	22,46	19,61	20,51	21,37
TAS2R46	42,38	38,46		33,83	33,12	29,88	28,43	23,12	19,28	22,81	24,45
Tas2r117	41,12	37,54	33,83		29,29	24,21	24,05	22,65	19,37	20,94	20,94
Tas2r105	30,82	28,49	33,12	29,29		26,14	28,21	23,26	20,72	22,74	21,50
Tas2r126	22,64	24,72	29,88	24,21	26,14		25,93	24,44	23,10	25,91	24,39
TAS2R1	24,93	23,21	28,43	24,05	28,21	25,93		23,70	25,23	20,69	21,32
TAS2R39	23,33	22,46	23,12	22,65	23,26	24,44	23,70		47,93	28,45	26,76
Tas2r144	20,52	19,61	19,28	19,37	20,72	23,10	25,23	47,93		27,95	25,23
TAS2R4	20,54	20,51	22,81	20,94	22,74	25,91	20,69	28,45	27,95		66,56
Tas2r108	21,43	21,37	24,45	20,94	21,50	24,39	21,32	26,76	25,23	66,56	

Supplementary Figure 13: Amino acid sequence identities of human and mouse bile acid-sensitive receptors. The pairwise amino acid sequence identities of the indicated bitter taste receptors in % were determined with CLC Main Workbench 22.0.2.

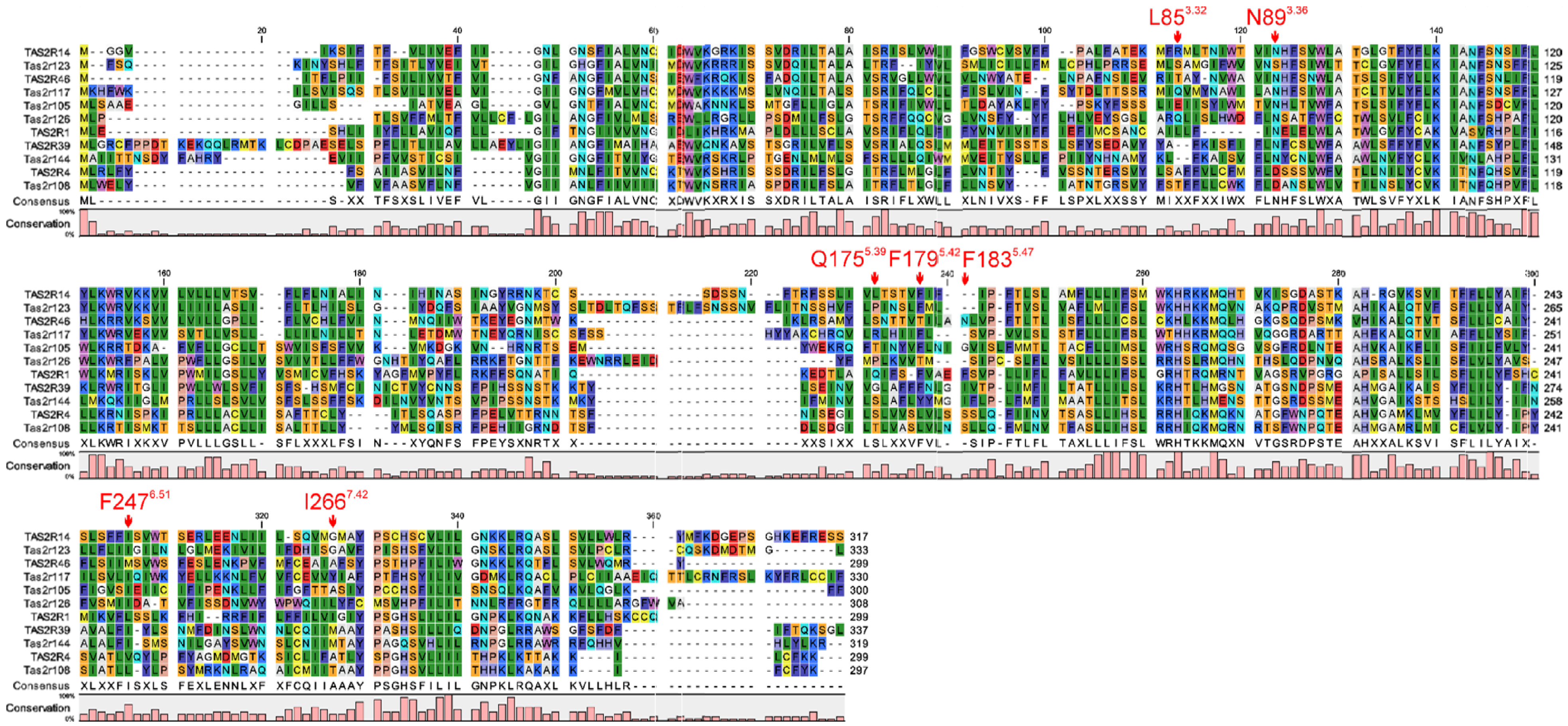

Supplementary Figure 14: Amino acid sequence alignment of human and mouse bile acid-sensitive receptors. The alignment was done using CLC Main Workbench 22.0.2. The color code is provided according to ClustalW conservation code. Positions indicated by red arrows refer to bile acid interacting positions in TAS2R1.

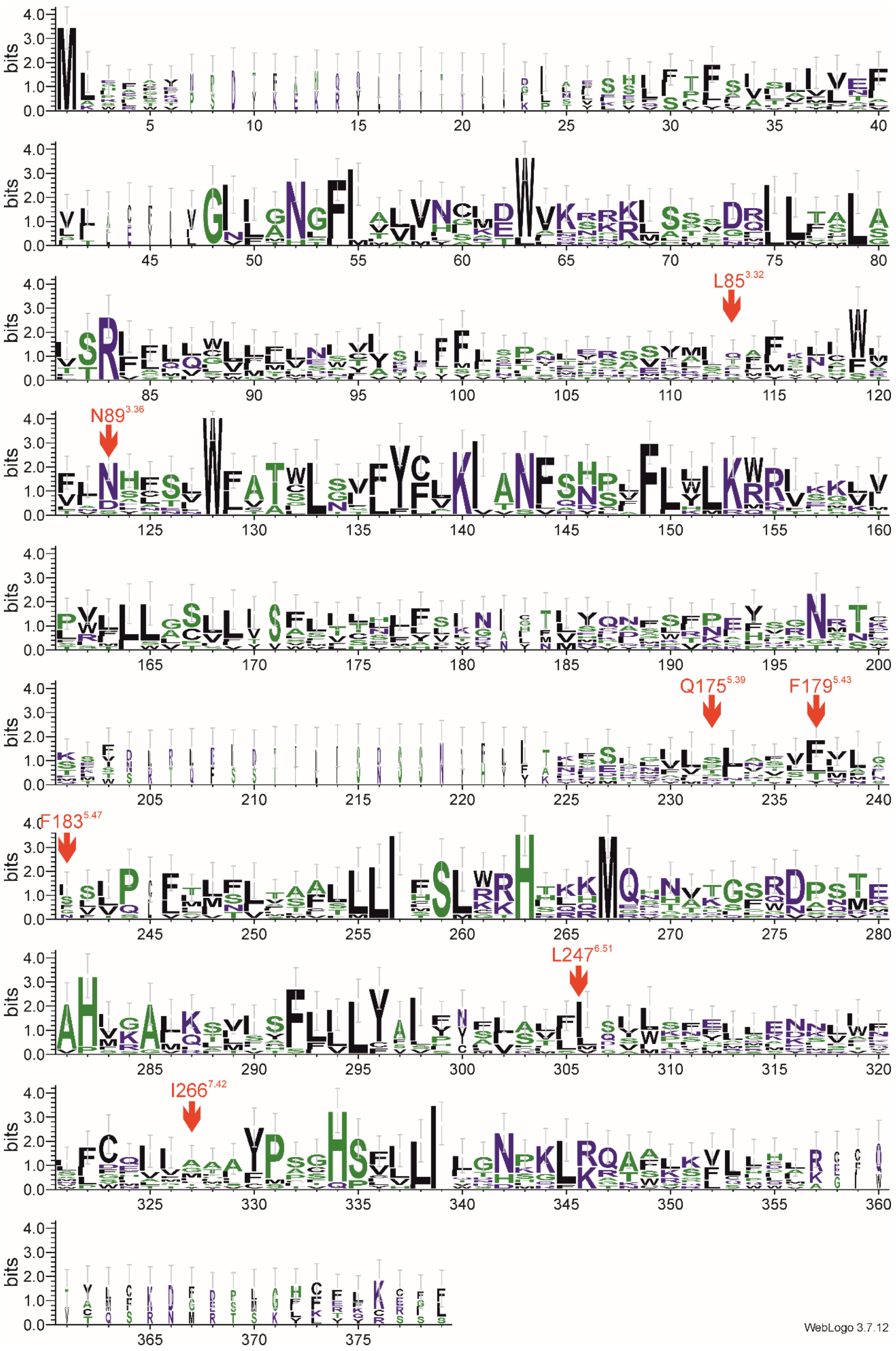

Supplementary Figure 15: WebLogo depiction of the amino acid sequence alignment (done with CLC Main Workbench 22.0.2) of bile acid-sensitive human and mouse bitter taste receptors. Positions indicated by red arrows refer to bile acid interacting positions in TAS2R1. WebLogo was created with the Web Logo 3 tool (https://weblogo.threeplusone.com).

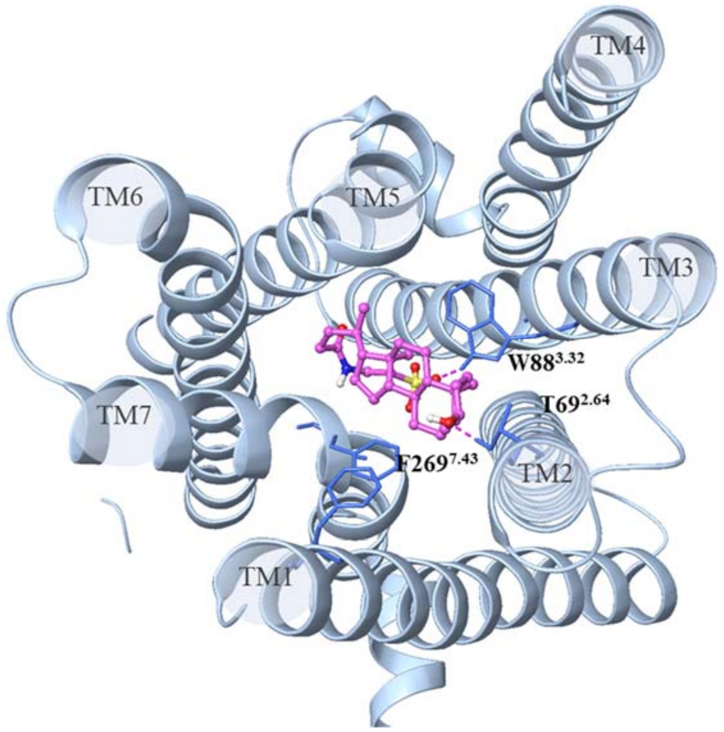

Supplementary Figure 16: 3D representation of the putative binding mode of taurolithocholic acid in the TAS2R46 (PDB ID: 7XP5) binding site obtained with MM/GBSA refinement (score: -70.21 kcal/mol).

Supplementary Table

Supplementary Table 1: Calculated EC_{50}-values of human and murine bitter taste receptors activated by bile acids.

	TAS2R1	TAS2R46	Tas2r108	Tas2r117
**Cholic Acid**	-	-	-	20.6 ± 5.1 µM
**Chenodeoxycholic Acid**	-	-	-	-
**Lithocholic Acid**	0.9 ± 0.1 µM	-	-	-
**Deoxycholic Acid**	6.1 ± 0.6 µM	-	-	-
**Taurocholic Acid**	-	-	-	37.4 ± 7.3 µM
**Glycocholic Acid**	-	-	-	16.0 ± 2.5 µM
**Taurolithocholic Acid**	1.9 ± 0.9 µM	1.7 ± 0.2 µM	2.3 ± 1.8 µM	-
**Ursodeoxycholic Acid**	-	-	-	-
