# Supplementary material for: Physiological activation of human and mouse bitter taste receptors by bile acids
Source: Commun Biol. 2023 Jun 7;6:612. doi: 10.1038/s42003-023-04971-3 (PMC10247784; doi:10.1038/s42003-023-04971-3)
Supplement: Supplementary file 2 — Description of Additional Supplementary Files [file 42003_2023_4971_MOESM2_ESM.pdf]

Description of Additional Supplementary Files

File name: Supplementary Data 1

Description: The source data for figures 2 and 3

File name: Supplementary Data 2

Description: The source data for figures 4 and 6

File name: Supplementary Data 3

Description: The source data for figure 5
